# Supplementary material for: Genetic Variation Underpinning ADHD Risk in a Caribbean Community
Source: Cells. 2019 Aug 16;8(8):907. doi: 10.3390/cells8080907 (PMC6721689; doi:10.3390/cells8080907)
Supplement: Supplementary file 1 [file cells-08-00907-s001.pdf]

cells

Genetic Variation Underpinning ADHD Risk in a Caribbean Community

Pedro J. Puentes-Rozo[1,2,*], Johan E. Acosta-López[1], Martha L. Cervantes-Henríquez[1,3], Martha L. Martínez-Banfi[1], Elsy Mejia-Segura[1], Manuel Sánchez-Rojas[1], Marco E. Anaya-Romero[4], Antonio Acosta-Hoyos[4], Guisselle A. García-Llinás[3], Claudio A. Mastronardi[5], David A. Pineda[6,7], F. Xavier Castellanos[8,9], Mauricio Arcos-Burgos[10,*,#], Jorge I. Vélez[3,*,#]

[1] Grupo de Neurociencias del Caribe, Unidad de Neurociencias Cognitivas, Universidad Simón Bolívar, Barranquilla, Colombia. ppuentes1@unisimonbolivar.edu.co (P.J.P.R); jacosta@unisimonbolivar.edu.co (J.E.A.L); cervantesmh@unisimonbolivar.edu.co (M.L.C.H); mmartinez108@unisimonbolivar.edu.co (M.L.M.B); emejia18@unisimonbolivar.edu.co (E.M.S); sanchezr@unisimonbolivar.edu.co (M.S.R).

[2] Grupo de Neurociencias del Caribe, Universidad del Atlántico, Barranquilla, Colombia.

[3] Universidad del Norte, Barranquilla, Colombia. jvelezv@uninorte.edu.co (J.I.V); gagarcia@uninorte.edu.co (G.A.G.L)

[4] Grupo de Investigación en Genética, Laboratorio de Genética y Biología Molecular, Universidad Simón Bolívar, Barranquilla, Colombia. manaya6@unisimonbolivar.edu.co (M.E.A.R); aacosta24@unisimonbolivar.edu.co (A.A.H)

[5] INPAC Research Group, Fundación Universitaria Sanitas, Bogotá, Colombia. mastronardic@hotmail.com

[6] Neuroscience Research Group, University of Antioquia, Medellín, Colombia. david.pineda1@udea.edu.co

[7] Neuropsychology and Conduct Research Group, University of San Buenaventura, Medellín, Colombia.

[8] Department of Child and Adolescent Psychiatry, Hassenfeld Children's Hospital at NYU Langone, New York, NY, USA. francisco.castellanos@nyumc.org

[9] Nathan Kline Institute for Psychiatric Research, Orangeburg, NY, USA.

[10] Grupo de Investigación en Psiquiatría (GIPSI), Departamento de Psiquiatría, Instituto de Investigaciones Médicas, Facultad de Medicina, Universidad de Antioquia, Medellín, Colombia. mauricio.arcos@udea.edu.co

[*] These authors contributed equally to this work.

[#] Correspondence: mauricio.arcos@udea.edu.co (M.A.B); jvelezv@uninorte.edu.co (J.I.V)

Supplementary Material:

Table S1. Single nucleotide polymorphisms (SNPs) genotyped in 386 individuals belonging to 113 nuclear families from Barranquilla, Colombia.

Marker	Alleles	Chr	Position	Closest gene(s)	QC result	Observations
rs143020947	A/G	2	143,643,356	*KYNU*	NO PASS	Monomorphic
rs207462497	G/T	2	143,689,722	*KYNU*	NO PASS	Monomorphic
rs1565902	C/T	4	62,091,215	*ADGRL3*	PASS	-
rs10001410	A/C	4	62,156,824	*ADGRL3*	PASS	-
rs2122642	G/A	4	62,380,859	*ADGRL3*	PASS	-
rs6551660	C/G	4	62,390,745	*ADGRL3*	NO PASS	HW criterion
rs207464763	A/G	4	62,416,514	*ADGRL3*	NO PASS	Monomorphic
rs1510921	C/T	4	62,578,187	*ADGRL3*	NO PASS	HW criterion
rs6551678	A/G	4	62,705,646	*ADGRL3*	NO PASS	HW criterion
rs207464765	A/T	4	62,720,720	*ADGRL3*	NO PASS	Monomorphic
rs748519607	A/T	4	63,888,775	*ADGRL3*	NO PASS	HW criterion
rs28363183	G/A	5	1,444,398	*SCL6A3*	NO PASS	Monomorphic
rs2282794	A/G	5	141,961,893	*FGF1*	PASS	-
rs761010945	A/C	9	136,499,907	*DBH*	NO PASS	Monomorphic
rs569034387	C/G	9	136,500,791	*DBH*	NO PASS	Monomorphic
rs199926239	A/T	9	136,512,962	*DBH*	NO PASS	Monomorphic
rs916457	C/T	11	627,014	*DRD4*	PASS	-
rs1800443	G/T	11	629,830	*DRD4*	NO PASS	HW criterion
rs2236225	G/A	14	63,978,598	*MTHFD1*	NO PASS	Monomorphic
rs11568324	C/T	16	54,283,559	*SLC6A2*	NO PASS	MAF criterion
rs371002154	A/C	16	55,726,127	*SLC6A2*	NO PASS	Monomorphic
rs79777590	C/T	17	4,298,478	*UBE2G1/SPNS3*	NO PASS	Monomorphic
rs565399294	A/G	17	4,395,222	*SPNS3/SPNS2*	NO PASS	Monomorphic
rs2228130	C/T	17	7,345,715	*POLR2A*	NO PASS	MAF criterion
rs6108461	A/G	20	10,215,270	*SNAP25*	NO PASS	HW criterion
rs362990	A/T	20	10,224,221	*SNAP25*	PASS	-

[a] UCSC GRCh37/hg19 coordinates. Chr: Chromosome; QC: Quality control; HW: Hardy-Weinberg. Markers highlighted in **blue** passed quality control filters and were further included for analyses.
